# Profiling of pathogenic variants in Japanese patients with sarcoglycanopathy

**DOI:** 10.1186/s13023-024-03521-2

**Published:** 2025-01-04

**Authors:** Rui Shimazaki, Yoshihiko Saito, Tomonari Awaya, Narihiro Minami, Ryo Kurosawa, Motoyasu Hosokawa, Hiroaki Ohara, Shinichiro Hayashi, Akihide Takeuchi, Masatoshi Hagiwara, Yukiko K. Hayashi, Satoru Noguchi, Ichizo Nishino

**Affiliations:** 1 Department of Neuromuscular Research, National Institute of Neuroscience, National Center of Neurology and Psychiatry, 4-1-1 Ogawa-Higashi, Kodaira, Tokyo, 187-8502 Japan; 2 Department of Genome Medicine Development, Medical Genome Center, National Center of Neurology and Psychiatry, Tokyo, Japan; 3 Department of Anatomy and Developmental Biology, Graduate School of Medicine, Kyoto University, Kyoto, Japan; 4 Center for Anatomical Studies, Graduate School of Medicine, Kyoto University, Kyoto, Japan; 5 Department of Developmental Biology and Functional Genomics, Ehime University Graduate School of Medicine, Ehime, Japan; 6 Department of Pathophysiology, Tokyo Medical University, Tokyo, Japan

**Keywords:** Sarcoglycanopathies, Haplotyping, Japan, Genetic Profile, RNA-Seq, Limb-Girdle muscular dystrophies, Variants, Large deletion, Skeletal muscles, in silico prediction

## Abstract

**Background:**

Sarcoglycanopathies (SGPs) are limb-girdle muscular dystrophies (LGMDs) that can be classified into four types, LGMDR3, LGMDR4, LGMDR5, and LGMDR6, caused by mutations in the genes, *SGCA*, *SGCB*, *SGCG*, and *SGCD*, respectively. SGPs are relatively rare in Japan. This study aims to profile the genetic variants that cause SGPs in Japanese patients.

**Methods:**

Clinical course and pathological findings were retrospectively reviewed in Japanese patients with SGP. Genetic analyses were performed using a combination of targeted resequencing with a hereditary muscle disease panel, whole genome sequencing, multiplex ligation-dependent probe amplification, and long-read sequencing. The structures of transcripts with aberrant splicing were also determined by RT-PCR, RNA-seq, and in silico prediction.

**Results:**

We identified biallelic variants in SGC genes in 53 families, including three families with LGMDR6, which had not been identified in Japan so far. *SGCA* was the most common causative gene, accounting for 56% of cases, followed by *SGCG*, *SGCB*, and *SGCD*, at 17%, 21%, and 6%, respectively. Missense variants in *SGCA* were very frequent at 78.3%, while they were relatively rare in *SGCB*, *SGCG*, and *SGCD* at 11.1%, 18.2%, and 16.6%, respectively. We also analyzed the haplotypes of alleles carrying three variants found in multiple cases: c.229C > T in *SGCA*, c.325C > T in *SGCB*, and exon 6 deletion in *SGCG*; two distinct haplotypes were found for c.229C > T in *SGCA*, while each of the latter two variants was on single haplotypes.

**Conclusions:**

We present genetic profiles of Japanese patients with SGPs. Haplotype analysis indicated common ancestors of frequent variants. Our findings will support genetic diagnosis and gene therapy.

**Supplementary Information:**

The online version contains supplementary material available at 10.1186/s13023-024-03521-2.

## Background

Sarcoglycanopathies (SGPs) are autosomal recessive limb-girdle muscular dystrophies (LGMDs) caused by mutations in four genes: *SGCA*, *SGCB*, *SGCG*, and *SGCD*, which lead to α-SGP (LGMDR3), β-SGP (LGMDR4), γ-SGP (LGMDR5), and δ-SGP (LGMDR6), respectively [1]. Since the protein products of *SGCA*, *SGCB*, *SGCG*, and *SGCD* form a sarcoglycan complex and work together, the symptoms among SGPs are almost similar. However, there are slight differences. For instance, LGMDR4 are more likely to be associated with cardiomyopathy, and LGMDR3 may have a milder symptom and slightly later onset and slower progression [2, 3].

Genetic analyses of patients with SGP from Algeria, China, and Europe have been reported [2–4], demonstrating that many share the same variants, and that alleles harboring variants are carried on specific haplotypes, indicating founder effects underlying SGPs in various countries [5–8]. In contrast, SGPs are relatively rare in Japan, although there have been a few reports of patients with LGMDR3, LGMDR4, or LGMDR5 [9–11].

Previous genetic research into SGPs has focused on variants within exons and their flanking intronic regions; however, technological advances in analysis techniques, including multiplex ligation-dependent probe amplification (MLPA), RNA-seq, and long-read sequencing, have enabled identification of genetic variations in intronic regions, as well as structural genomic changes [12]. Further, the development of artificial intelligence-based prediction tools has contributed to more accurate and cost-effective identification of splice changes [13]. The aims of this study were to profile the genetic variation in Japanese patients with SGP and identify variant founder effects.

## Methods

### Patients

This retrospective cohort study was performed using samples and datasets sent to the National Center of Neurology and Psychiatry between January 1979 and June 2023. Cases with absent or reduced expression of sarcoglycan, detected by immunohistochemistry, and biallelic variants in *SGCA*, *SGCB*, *SGCG*, or *SGCD*, were selected for inclusion in these analyses. This study cohort included 39 patients with negative Western blotting result for *SGCA*.

### Muscle biopsy

Muscle samples for histological examination were frozen in isopentane cooled with liquid nitrogen. A series of histochemical and immunohistochemical analyses, including assessment of sarcoglycan,　were performed for diagnosis. Routine histochemical and immunohistochemical stainings were conducted using standard procedures [14].

### Genetic analysis

Genomic DNA was extracted from peripheral blood or muscle samples from patients, using standard procedures [15]. All exons and flanking intron regions of *SGCA*, *SGCB*, *SGCG*, and *SGCD* were amplified; some cases were also analyzed by whole genome sequencing. All variants were confirmed by Sanger sequencing. MLPA was conducted to analyze suspected large deletions, as previously described [16], using SALSA MLPA Probemix P116 SGC (MRC Holland, Amsterdam, The Netherlands), according to the manufacturer’s instructions.

### RNA-seq

We applied RNA-seq for 2 patients. As previously reported [12], total RNA was extracted from frozen muscle using the RNAeasy mini kit (QIAGEN, Valencia, CA, USA) according to the manufacturer’s instructions. Libraries were prepared from total RNA using Ribo-Zero Gold (Illumina), according to the manufacturer’s instructions, and 100 bp paired-end sequencing was performed on the NovaSeq 6000 platform (Macrogen, Seoul, Korea). RNA-seq reads were mapped using Spliced Transcripts Alignments to Reference software (https://github.com/alexdobin/STAR).

### RT-PCR

We confirmed the splicing abnormality by RT-PCR for 5 patients. Total RNA was extracted from frozen muscle samples using an RNAeasy mini kit. Complementary DNA was generated by reverse transcription of RNA using SuperScript IV VILO Master Mix (ThermoFisher Scientific, Waltham, MA, USA). Primers are listed in Table S1 (see Additional file). The sequences of the products were confirmed by Sanger sequencing.

### In silico* prediction*

The pathogenicity of missense variant is predicted to be pathogenic by more than one in silico tool (PolyPhen-2 (http://genetics.bwh.harvard.edu/pph2/), MutationTaster (http://www.mutationtaster.org/), or CADD (http://cadd.gs.washington.edu/)). The criteria for pathogenicity by prediction tool include ‘Probably Damaging’ for PolyPhen-2, ‘Disease-causing’ for MutationTaster, and a CADD score of ≥ 20. SpliceAI [13] and MaxEntScan [17] were used to predict the effects of identified single nucleotide variants (SNVs) on aberrant splicing. SpliceAI calculates the changes in activities of splicing sites (AG, gain of acceptor site; AL, loss of acceptor site; DG, gain of donor site; DL, loss of donor site) as delta scores, where higher scores indicate that SNVs are more likely to alter splicing. MaxEntScan assigns a log odds ratio (MaxEnt score) to the sequences of 5' and 3' splice sites, where higher scores indicate higher probabilities that splice sites are used. PDIVAS was used to predict pathogenicity of deep-intronic variants [18]. To verify the protein 3D structure, we applied the software AlphaFold (https://alphafold.ebi.ac.uk/). We also applied the prediction software iPSORT (https://ipsort.hgc.jp/) and WoLF PSORT (https://wolfpsort.hgc.jp/) to predict the subcellular localization of protein based on amino acid sequencing.

### Nanopore CRISPR/Cas9-targeted long-read resequencing

Long-read sequencing was performed using genomic DNA extracted from patient blood. Library preparation was performed using a Ligation Sequencing Kit (SQK-LSK109; Oxford Nanopore Technologies Inc., Oxford, UK) with CRISPR-Cas9. Guide RNA (crRNA) sequences are listed in Table S2 (see Additional file). Prepared libraries were applied to MinION flow cells and sequenced on an MK1C instrument (Oxford Nanopore Technologies Inc., Oxford, UK). Real-time base calling was performed using MinKnow integrated in Guppy. Sequences were mapped to the human genome reference sequence (GRCh38) using Minimap2 (https://github.com/lh3/minimap2).

### Haplotype analyses

Single nucleotide polymorphisms (SNPs) around the variants, c.229C > T in *SGCA*, c.325C > T in *SGCB*, and exon 6 deletion in *SGCG*, were selected as neutral markers for haplotype reconstruction, based on their genomic position and allele frequency (< 0.2). Primers are shown in Table S3 (see Additional file).

## Results

**Fig. 1:**
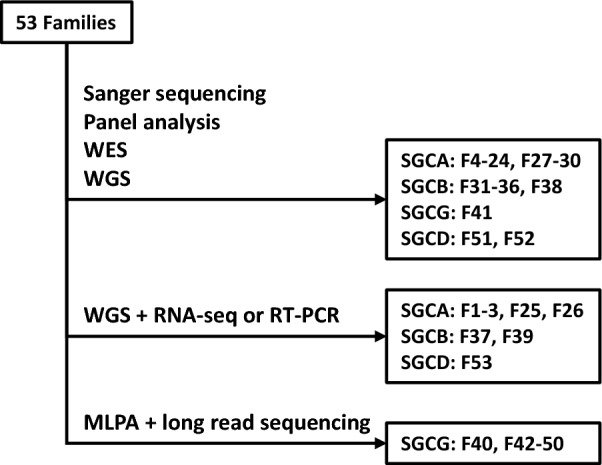
STRAD flow diagram showing the genetic analysis carried out. We started with sequencing analyses, such as Sanger sequencing, panel analysis, whole-exome sequencing, or whole-genome sequencing. Aberrant splicing was detected using RNA-seq or RT-PCR in patients with exon–intron boundary or intronic variants. MLPA was applied to patients who were not diagnosed by sequencing analysis, and the breakpoints of a large *SGCG* deletion were identified using long-read sequencing

**Fig. 2:**
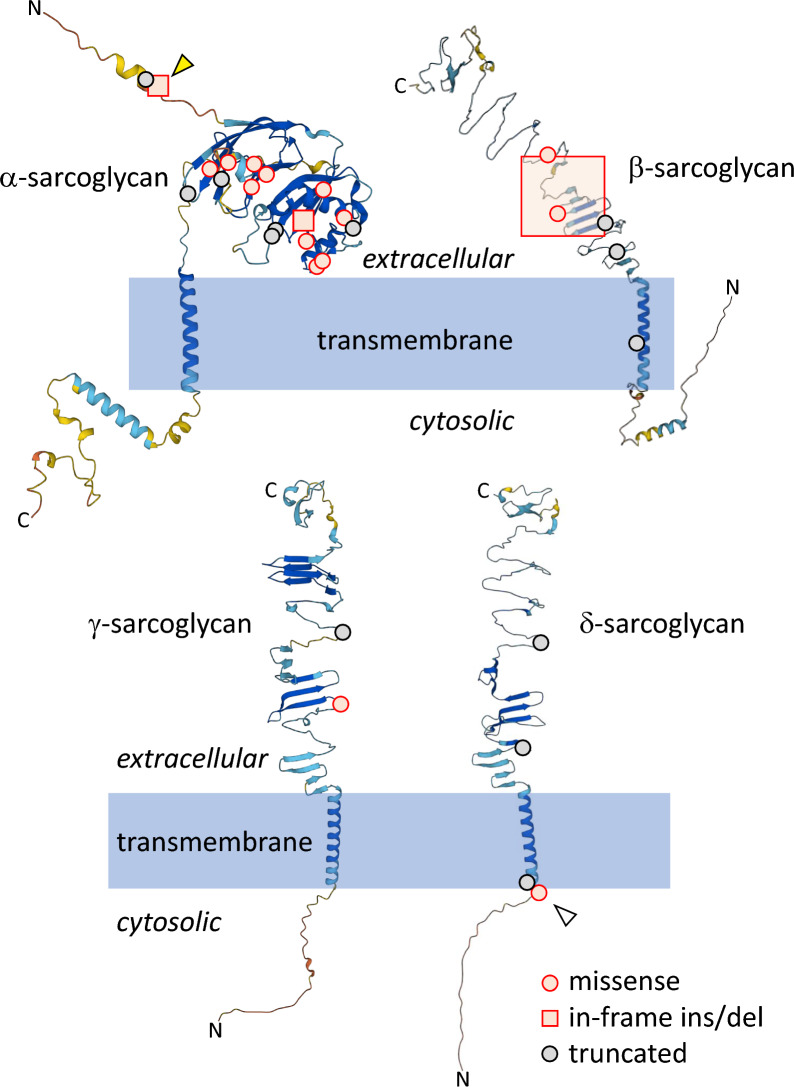
Locations of amino-acid changes caused by identified genetic variants in predicted protein structures. Predicted three-dimensional structures of α-sarcoglycan (Q16586 (SGCA_HUMAN)), β-sarcoglycan (Q16585 (SGCB_HUMAN)), γ-sarcoglycan (Q13326 (SGCG_HUMAN)), and δ-sarcoglycan (Q92629 (SGCD_HUMAN)), obtained from Alphafold Protein Structure Database (https://alphafold.ebi.ac.uk/). Red circles indicate the positions of missense variants. Red squares indicate in-frame deletions or insertions. Gray circles denote the positions of truncation of normal sequences in truncation variants. Yellow and white arrowheads show the 4 amino-acid deletion (13–16) caused by c.38-2A > C in *SGCA* and p.K32E encoded by c.94A > G in *SGCD*, respectively

**Fig. 3:**
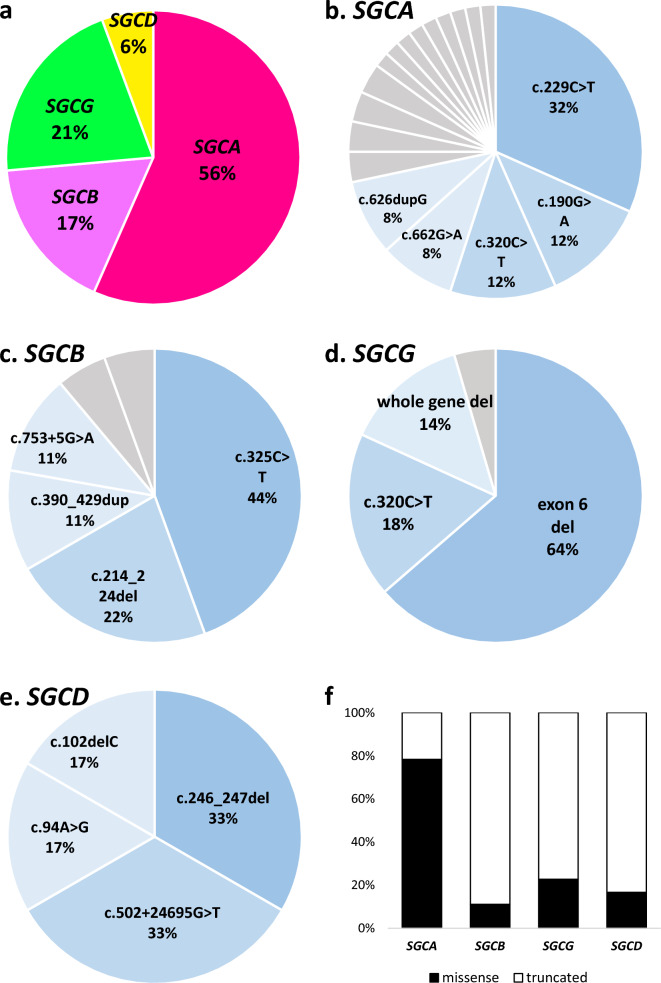
Frequencies of variants and variant types in patients with sarcoglycanopathies. The frequency of (**a**) causative genes in Japanese patients with sarcoglycanopathies, and (**b**)–(**e**) variants in each causative gene: **b** *SGCA*, **c** *SGCB*, **d** *SGCG*, and **e** *SGCD*. Gray sections in pie charts indicate variants detected one family. **f** Effects of gene variants on protein products

We identified 53 Japanese families (Fig. 1) and 55 patients who harbored biallelic variants in any of the *SGC* genes analyzed (Table 1 and Fig. 2). Variants in *SGCA*, *SGCB*, *SGCG*, and *SGCD* were identified in 30 (56%), 9 (17%), 11 (21%), and 3 (6%) families, respectively (Fig. 3a). Among 32 identified variants, 14 had previously been reported and 18 were novel. In previous reports, limited types of variants have been identified in the different *SGC* genes, regardless of the causative gene involved and in the current study, we identified 18, 6, 4, and 4 types of variants in *SGCA*, *SGCB*, *SGCG* and *SGCD*, respectively. Some recurrent variants were identified in multiple patients (Fig. 3b–d). Notably, allele frequencies of the most common variants in *SGCA*, *SGCB*, and *SGCG* in our study population reached 32%–64%.

**Table 1:** Variants found in Japanese patients with sarcoglycanopathy

Family	Age at onset	Age at biopsy	Sex	Serum CK (UI/L)	Symptoms	Causative gene	Variants
F1	2y	4y	F	5393	High CK	*SGCA*	c.37 + 6 T > C [41] c.725 T > C, p.V242A[42]
F2	5 y	5 y	M	4538	High CK	*SGCA*	**c.38-2A > C** c.320C > T, p.A107V[43]
F3	4 y	37 y	F	206	Arm and leg weakness, respiratory failure	*SGCA*	**c.158-2_167del** c.626dupG, p.C209Wfs*10
F4	58 y	59 y	M	14620	Muscle pain	*SGCA*	c.190G > A, p.A64T (homozygous)[44]
F5	50 y	72 y	F	1141	Arm and leg weakness, calf hypertrophy	*SGCA*	c.190G > A, p.A64T (homozygous)
F6	3 y	56 y	M	No data	Leg weakness, respiratory failure, heart failure	*SGCA*	c.190G > A, p.A64T (homozygous)
F7	9 y	42 y	M	2630	Arm and leg weakness	*SGCA*	c.190G > A, p.A64T c.626dupG, p.C209Wfs*10
F8	6 y	18 y	F	706	Leg weakness, calf hypertrophy	*SGCA*	c.220C > T, p.R74W (homozygous)[45]
F9	3 y	12 y	M	7244	Leg weakness, calf hypertrophy	*SGCA*	c.229C > T, p.R77C (homozygous)[46]
F10	3 y	7 y	M	13299	Leg weakness, calf hypertrophy	*SGCA*	c.229C > T, p.R77C (homozygous)
F11	6 y	15 y	M	5159	Leg weakness, calf hypertrophy	*SGCA*	c.229C > T, p.R77C (homozygous)
F12	10 m	11 y	F	7581	Leg weakness, muscle pain, calf hypertrophy	*SGCA*	c.229C > T, p.R77C (homozygous)
F13	6 y	8 y	M	9000	Leg weakness, calf hypertrophy	*SGCA*	c.229C > T, p.R77C (homozygous)
F14	6 y	17 y	F	3746	Leg weakness	*SGCA*	c.229C > T, p.R77C (homozygous)
F15	1 y	9 y	M	8415	Arm and leg weakness	*SGCA*	c.229C > T, p.R77C (homozygous)
F16	2 y	6 y	F	2599	Muscle pain	*SGCA*	c.229C > T, p.R77C c.320C > T, p.A107V
F17	5 m	13 y	M	1775	Arm and leg weakness muscle pain, calf hypertrophy	*SGCA*	c.229C > T, p.R77C c.320C > T, p.A107V
F18	28 y	35 y	M	1357	Muscle pain	*SGCA*	c.229C > T, p.R77C c.320C > T, p.A107V
F19	2 y	2 y	F	6280	Equinus foot	*SGCA*	c.229C > T, p.R77C **c.409_410delinsCCTGGTGCGCAGCCAGG, p.E137delinsPGAQPG**
F20	2 y	2 y	F	13766	Equinus foot	*SGCA*	c.229C > T, p.R77C c.626dupG, p.C209Wfs*10
F21-1*	3 y	9 y	M	14418	Leg weakness, muscle pain, calf hypertrophy, equinus foot	*SGCA*	c.266 T > C, p.L89P[47] **c.749 T > G, p.V250G**
F21-2*	2 y	7 y	M	7455	Leg weakness, muscle pain		
F22	1 y	6 y	M	516	Muscle pain, equinus foot	*SGCA*	c.271G > A, p.G91S[48] c.320C > T, p.A107V
F23	6 y	48 y	M	1328	Muscle pain, trunk and leg weakness	*SGCA*	c.320C > T, p.A107V **c.409_410delinsCCTGGTGCGCAGCCAGG, p.E137delinsPGAQPG**
F24	62 y	70 y	F	1115	Leg weakness	*SGCA*	c.320C > T, p.A107V **c.662G > C, p.R221P**
F25	No data	71 y	F	No data	No data	*SGCA*	**c.584 + 1G > A** c.662G > A, p.R221H[49]
F26	6 y	54 y	M	887	Arm and leg weakness, calf hypertrophy	*SGCA*	c.584 + 5G > T (homozygous)[50]
F27	3 y	29 y	M	465	Arm and leg weakness, respiratory failure, heart failure	*SGCA*	c.626dupG, p.C209Wfs*10 (homozygous)
F28	6 y	80 y	F	342	Arm and leg weakness	*SGCA*	**c.634A > G, p.M212V (homozygous)**
F29	5 m	3 y	M	555	Calf hypertrophy	*SGCA*	c.662G > A, p.R221H (homozygous)[49]
F30	59 y	67 y	M	333	Arm and leg weakness	*SGCA*	c.662G > A, p.R221H (homozygous)
F31	1 y	2 y	M	42630	Leg weakness, calf hypertrophy	*SGCB*	c.214_221del, p.L72Pfs*24 (homozygous)
F32	4 y	11 y	M	8022	Leg weakness, calf hypertrophy, equinus foot	*SGCB*	c.214_221del, p.L72Pfs*24 c.325C > T, p.R109*[51]
F33-1*	3 y	3 y	M	3994	Leg weakness, equinus foot, intellectual disability	*SGCB*	c.214_221del, p.L72Pfs*24 c.325C > T, p.R109*
F33-2*	4 m	4 m	M	2420	High CK		
F34	1 y	7 y	F	9920	Arm and leg weakness, intellectual disability, respiratory failure	*SGCB*	c.325C > T, p.R109* (homozygous)
F35	9 m	10 m	F	32400	Muscle weakness, calf hypertrophy, equinus foot	*SGCB*	c.325C > T, p.R109* (homozygous)
F36	8 y	9 y	F	21944	Leg weakness, calf hypertrophy, equinus foot	*SGCB*	c.325C > T, p.R109* **c.607G > C, p.A203P**
F37	4 y	6 y	M	24880	Leg weakness, calf hypertrophy, equinus foot	*SGCB*	c.325C > T, p.R109* c.753 + 5G > A[52]
F38	3 y	4 y	M	3254	Calf hypertrophy, equinus foot	*SGCB*	c.390_429dup, p.I144Afs*3 (homozygous)
F39	3 y	7 y	M	3370	Leg weakness, intellectual disability	*SGCB*	c.499G > A, p.G167S[53] c.753 + 5G > A
F40	1 y	8 y	F	15772	Leg weakness, calf hypertrophy	*SGCG*	c.320C > T, p.S107L[2] **Exon 1–6 deletion**
F41	0 m	2 y	F	17770	Leg weakness, calf hypertrophy, equinus foot	*SGCG*	c.320C > T, p.S107L (homozygous)
F42	1 y	3 y	M	8557	Leg weakness, calf hypertrophy	*SGCG*	c.320C > T, p.S107L Exon 6 deletion[54]
F43	2 y	6 y	M	28300	Leg weakness, calf hypertrophy, equinus foot	*SGCG*	Exon 6 deletion (homozygous)
F44	4 y	4 y	M	10176	Leg weakness, calf hypertrophy	*SGCG*	Exon 6 deletion (homozygous)
F45	2 y	5 y	M	5524	Leg weakness, calf hypertrophy, equinus foot	*SGCG*	Exon 6 deletion (homozygous)
F46	6 y	34 y	F	1240	Leg weakness, calf hypertrophy, equinus foot	*SGCG*	Exon 6 deletion (homozygous)
F47	7 y	8 y	M	15336	Leg weakness	*SGCG*	Exon 6 deletion (homozygous)
F48	6 y	25 y	M	515	Leg weakness, calf hypertrophy, respiratory failure	*SGCG*	Exon 6 deletion Whole gene deletion[33]
F49	1 y	3 y	M	9760	Leg weakness, calf hypertrophy	*SGCG*	Exon 6 deletion Whole gene deletion
F50	1 y	1 y	F	18630	Calf hypertrophy	*SGCG*	Exon 6 deletion Whole gene deletion
F51	2 y	2 y	F	17391	Calf hypertrophy, respiratory failure	*SGCD*	**c.94A > G, p.K32E** c.246_247del, p.S83*
F52	4 y	7 y	F	3290	Leg weakness, calf hypertrophy, equinus foot	*SGCD*	c.102delC, p.L35Cfs*9 c.246_247del, p.S83*
F53	6 y	44 y	F	1235	Leg weakness, intellectual disability	*SGCD*	**c.502 + 24695G > T (homozygous)**

Bold characters indicate novel variants. y, years; m, months; CK, creatinine kinase

*F21-1 and F21-2, and F33-1 and F33-2 are two pairs of siblings

Two patients with a homozygous c.220C > T variant, the most common variant in the *SGCA*, tended to manifest symptoms in childhood and lost ambulation. However, 5 patients carrying a c.220C > T variant in combination with another variant exhibited milder symptoms, such as myalgia or elevated CK levels, compared to the homozygous patients. Patients with the c.190G > A, c. 320C > T and c.662G > A variants in the *SGCA* exhibited relatively mild symptoms like only high CK levels, maintaining ambulation into advanced age. All patients with the variants in the *SGCB* and *SGCG* showed childhood-onset. Adult patients with whole deletion of *SGCG* gene had lost ambulation and required respiratory support. A patient carrying the c.246_247del variant in the *SGCD* required respiratory support from an early age, while a patient with c.502 + 24695G > T variants preserved ambulation but presented with comorbidities such as intellectual disabilities.

**Fig. 4:**
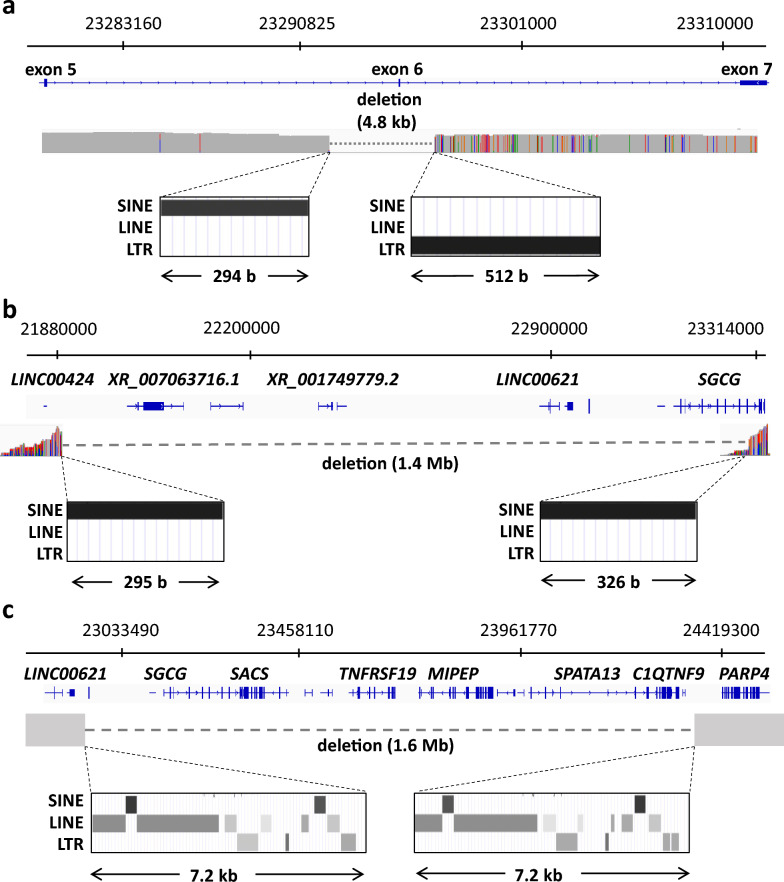
Genome structures in patients with deletion of *SGCG* exon 6, *SGCG* exon 1 to 6 and the whole *SGCG* gene. **a** *SGCG* exon 6 deletion. The breakpoints were the same in all patients. **b** *SGCG* exon 1 to 6 deletion. **c** Whole gene deletion of *SGCG*; similar sequences (identity: 94%) were present in the 5' and 3' breakpoints. Repeat sequence schema were retrieved from Ensembl (https://asia.ensembl.org/index.html). SINE, short interspersed nuclear element; LINE, long interspersed nuclear element; LTR, long terminal repeat

We identified three kinds of large deletions of the *SGCG* gene in nine families by MLPA, including deletions of exon 6, exon 1 to 6 and the entire gene (Table 1). To more accurately delineate the deleted regions, we applied Nanopore CRISPR/Cas9-targeted long-read resequencing. Genomic DNA samples from individuals 42–46 (F42-46) had deletions of precisely the same chromosome 13 region (Chr13: 3292402–23297276), which included exon 6 of *SGCG* (Fig. 4a). The 5'-breakpoint was within a short interspersed nuclear element (SINE) and the 3' breakpoint was within a long terminal repeat (LTR). In F40, 1.4-Mb sequence from downstream of *LINC00424* to intron 6 of *SGCG* (surrounding chr13: 23310090) was deleted. Both 5’- and 3’- breakpoints were within SINE (Fig. 4b). In F49, a 1.6 Mb sequence from downstream of *LINC00621* to upstream of *PPAR4*, including the whole *SGCG* gene, was deleted. Similar sequences (approximately 7.3 kb) containing repeat elements (SINE, LTR, and long interspersed nuclear elements) were present in sequences flanking both ends of the 1.6 Mb deletion; therefore, we could not determine the exact breakpoints (Fig. 4c). In other cases, who had entire *SGCG* deletion, long genomic DNAs, which were required for Nanopore CRISPR/Cas9-targeted long-read resequencing, were unavailable.

**Fig. 5:**
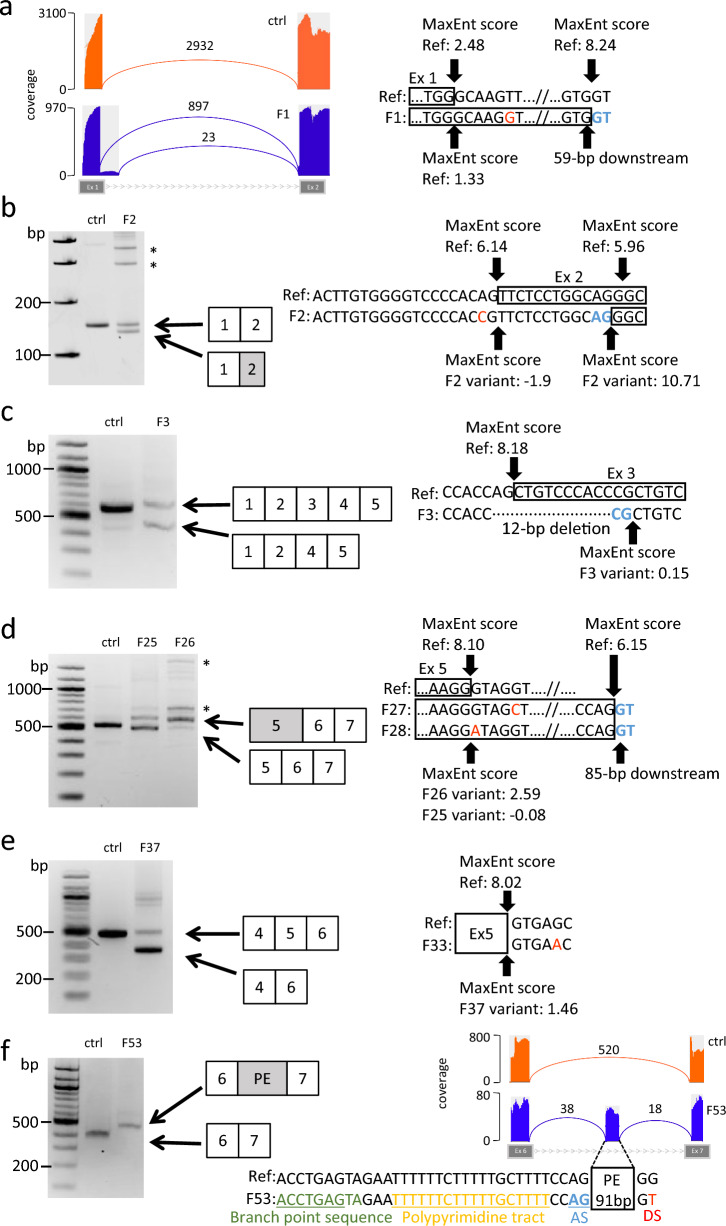
Splicing abnormalities and MaxEnt scores generated by analysis of intronic variants. **a** F1 *SGCA* intron 1: c.37 + 6 T > C. Sashimi plots of RNA-seq data using control and patient muscles are shown. **b**–**e** RT-PCR products obtained from control and patient muscle samples separated on agarose gels and schema showing aberrant splicing events with MaxEnt scores. White squares are normal-sized exons and gray squares are altered-sized exons. In the right schema, genetic variants are shown in red and new splicing sites are shown in light blue. * indicate the non-specific products which were not related to *SGC* genes. **b** F2 *SGCA* intron 1: c.38-2A > C. **c** F3 *SGCA* intron 2/exon 3: c.158-2_167del. **d** F25 *SGCA* intron 5: c.584 + 1G > A and F26 *SGCA* intron 5: c.584 + 5G > T. **e** F37 *SGCB* intron 5: c.753 + 5G > A. **f** F53 *SGCD* intron 6: c.502 + 24695G > T. RT-PCR and Sashimi plots of RNA-seq data are shown; sequences 5' (upstream) of the variants included a possible branch point site, a polypyrimidine tract, and a splice acceptor site. ctrl, control sample; Ref, reference sequence

Intronic single nucleotide variants were identified in five, two, and one families with LGMDR3, LGMDR4, and LGMDR6, respectively (Table 1) and we analyzed skeletal muscle transcripts from these patients to search for splicing abnormalities. We found a 59 bp extension to the 3’ end of exon 1 by RNA-seq in F1, since we could not obtain abnormal product by RT-PCR (Fig. 5a). One additional band from *SGCA* transcript was detected with some additional non-specific bands denoted by * in each of F2 and F3 by RT-PCR with primer pairs targeting *SGCA* exons 1–2 and 1–5, respectively; both products were amplified at comparable levels to those of the normal transcript. In The abnormal PCR product detected in F2 was truncated by 12 bp at the 5' end of exon 2, while that detected in F2 skipped exon 3 (Fig. 5b and c). RT-PCR analysis of samples from F25 and F26 with primers targeting exon 5–7 of *SGCA* generated the same longer product, which contained an 85 bp extension to the 3' end of exon 5, with an additional non-specific band denoted by *, in addition to the normal amplicon (Fig. 5d). In F37, which carried an *SGCB* variant, a smaller PCR product lacking exon 5, amplified at a comparable level to that of the normal product in control samples, was obtained by amplification of exon 4–6 (Fig. 5e). A deep intronic variant in *SGCD* was identified in F53 and found to result in transcription of a pseudoexon containing 91 bp of intron 6 (Fig. 5f).

To evaluate the pathogenicity of the identified intronic variants as determinants of aberrant splicing, we calculated the scores of the splicing acceptor and donor sites of affected exons using SpliceAI and MaxEntScan in silico (Table 2; Fig. 5a–f). In F1, F2, F3, F25, F26, and F37, losses of splice sites near the genetic variants were predicted by SpliceAI. Similarly, the scores for those sites were greatly reduced in MaxEntScan analysis. Both software packages predicted the creation of alternative splice sites, including a splice acceptor site 12-bp downstream of the normal site in F2 and a splice donor sites, 59-bp downstream in F1, and 85-bp downstream in F25 and F26, with high delta scores for AG and DG, respectively. SpliceAI and MaxEntScan also successfully predicted the pseudoexon-creation observed in F53, with high scores for donor and acceptor site creation (DG = 1.00 and AG = 0.95) in SpliceAI, and a high score of 9.31 at the new donor site using MaxEntScan against 1.67 of the reference sequence. In addition, the pathogenicity of the pseudo-exon creation was also predicted by PDIVAS with a high score of 0.971 [18]. Interestingly, this pseudoexon preserved the functional upstream sequence in the flanking intron; a branch-point-like structure at –29 nucleotides (nt), a polypyrimidine track at –22 to –5 nt, and a pseudoexon acceptor site. Together with the results of RT-PCR, these in silico predictions indicated that all variants identified in introns were pathogenic and the causes of aberrant splicing.

**Table 2:** Summary of splicing abnormalities and SpliceAI predictions

Family	Gene	Variant	Effect on transcript	Acceptor loss	Donor loss	Acceptor gain	Donor gain	ORF
F1	*SGCA*	Intron 1: c.37 + 6 T > C	Exon 1 extension	0	0.51	0	0.25	Out of frame
F2	*SGCA*	Intron 1: c.38-2A > C	Exon 2 shortening	0.96	0	0.68	0.09	In frame
F3	*SGCA*	Intron 2/exon 3: c.158-2_167del	Exon 3 skipping	0.99	0	0.49	0.01	Out of frame
F25	*SGCA*	Intron 5: c.584 + 1G > A	Exon 5 extension	0	0.99	0.01	0.54	Nonsense codon
F26	*SGCA*	Intron 5: c.584 + 5G > T	Exon 5 extension	0	0.58	0.01	0.48	Nonsense codon
F37	*SGCB*	Intron 5: c.753 + 5G > A	Exon 4 skipping	0	0.86	0.02	0	In frame
F53	*SGCD*	Intron 6: c.502 + 24695G > T	Pseudoexon creation	0	0	0.95	1.00	Nonsense codon

ORF, open reading frame

**Fig. 6:**
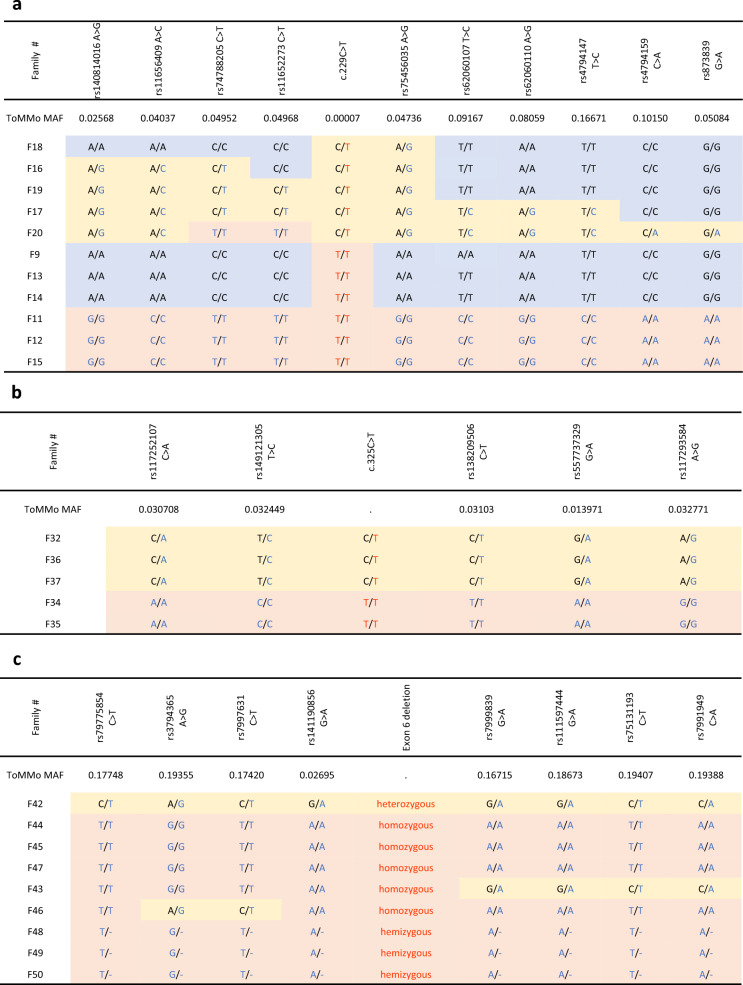
Haplotype analyses of alleles containing common single nucleotide polymorphism (SNP) variants. **a** Patients with *SGCA* c.229C > T. **b** Patients with *SGCB* c.325C > T. **c** Patients with *SGCG* exon 6 deletion. Bases in blue font indicate minor SNP alleles and bases in red font indicate variants. Orange shaded cells indicate homozygosity for minor SNP alleles. Yellow shaded cells indicate heterozygosity for minor SNP alleles. Blue shaded cells indicate homozygosity for major SNP alleles

Founder effects in patients with SGPs worldwide have been suggested in several previous reports [8, 19–21]. Therefore, we analyzed the haplotypes containing the following variants: *SGCA*: c.229C > T, *SGCB*: c.325C > T, and *SGCG*: exon 6 deletion (Fig. 6a–c). We identified 5–10 SNPs in a homozygous state in individuals homozygous for the pathogenic variants, F11 and F12 with *SGCA* c.229C > T, F34 with *SGCB* c.325C > T, and F47 with *SGCG* exon 6 deletion. Among families with the *SGCA* c.229C > T variant, F11, F12, and F15 had the same haplotype, while F9, F13, and F14 had a distinct haplotype (Fig. 6), indicating the presence of at least two haplotypes. Heterozygous individuals had compatible haplotypes, but were not informative, because they were hybrid for both genotypes. Families homozygous for *SGCB* c.325C > T (F34 and F35) shared the same haplotype, and those heterozygous for this variant (F32, F36, and F37) also possessed the same haplotype, indicating a single haplotype carrying *SGCB* c.325C > T. In families with *SGCG* exon 6 deletion, the homozygous individuals, F44, F45, and F47, and those hemizygous for the deletion, F48–F50, had the same haplotype; further, one heterozygous individual, F42, also showed compatible results, while the homozygotes, F43 and F46, had haplotypes with evidence of genetic recombination.

Notably, the frequencies of missense and truncation variants differed among causative genes of SGPs, as reported previously [2–4]. Missense variants in *SGCA* were frequent at 78.3%, while they were relatively rare in *SGCB*, *SGCG*, and *SGCD*, at 11.1%, 18.2%, and 16.7% respectively. By contrast, truncation variants were common in *SGCB*, *SGCG*, and *SGCD.*

## Discussion

In this study, we conducted genetic profiling of 53 Japanese families with SGP. In Japan, *SGCA* was the most common causative gene, followed by *SGCG*, *SGCB*, and *SGCD*. The proportion of causative genes varies by region, with *SGCA* being the most common in Europe, the USA and Brazil [2, 3, 22], *SGCB* in Iran [6] and *SGCG* in Algeria and India [4, 23]. No patients with LGMDR6 have previously been reported in Japan; here, we report the first identification of Japanese patients with LGMDR6, with biallelic variants in *SGCD*. Although the reason for the low prevalence of LGMDR6 is unclear, it has been suggested that *SGCD* is expressed in the heart, and that individuals with variants in *SGCD* may die in utero [24]; however, no cardiac disease was observed in our patients with LGMDR6, although genetically undiagnosed siblings of F53 are recorded to have died of cardiac disease.

A few types of variants accounted for more than half of alleles in all causative genes. In particular, c.229C > T (R77C) in *SGCA*, which is also reported to be common in other countries, and has been suggested as a founder variant [3, 25–27]. Analysis of the genotypes of Japanese patients with c.229C > T indicated the presence of two distinct haplotypes, suggesting that *SGCA*: c.229C > T can be considered a recurrent variant. The alleles harboring c.325C > T in *SGCB* and exon 6 deletion in *SGCG*, shared common haplotypes and were likely each inherited from a single common ancestor. This concept is significant, as genomic screening that prioritizes founder and recurrent variants enhances diagnosis efficacy and provide a foundation for the development of gene therapies.

Interestingly, large deletions of *SGCG* are common in Japan, and *SGCG* contains numerous repetitive sequences in its introns, which may be prone to genetic recombination. The breakpoints of the Exon 6 deletion and Exon 1 to 6 deletion were within an LTR and SINE respectively, and the breakpoints flanking whole *SGCG* deletions contained similar repetitive elements, suggesting non-allelic homologous recombination as the likely mechanism underlying both types of deletion [28]. Additionally, individuals with deletion of the entire *SGCG* gene also lacked five other genes, among which *SACS* and *MIPEP* are reported to cause spastic ataxia and combined oxidative phosphorylation deficiency 31, respectively [29, 30]. Thus far, no patient with SGP and homozygous deletion of the entire *SGCG* gene has been identified, as combined oxidative phosphorylation deficiency is a very severe condition. MLPA combined with long-read sequencing proved useful for the diagnosis of these variants.

We also predicted the pathogenicity of detected intronic variants and altered transcript structures in silico, as well as successfully identifying their splicing abnormalities in all cases by RT-PCR and RNA-seq. The shifted PCR products detected in F2 and F37 had in-frame changes and were not subject to nonsense-mediated mRNA decay; therefore, they appeared to be expressed in comparable amounts to normal transcripts in control samples. In *SGCD*, creation of a pseudoexon deep in intron 6 is pathogenic. Exon-skipping therapy is an approved treatment for Duchenne muscular dystrophy [31] and recently, we developed a therapeutic strategy using branchpoint-targeted antisense oligonucleotides for patients with Fukuyama congenital muscular dystrophy caused by an *FKTN* transcript involving a pseudoexon [32]. Accurate diagnosis of SGP may also provide therapeutic opportunities.

We found that many of the *SGCA* variants detected were predicted to be missense and to cause amino-acid substitutions in the protein, while most *SGCB*, *SGCG*, and *SGCD* variants were predicted to cause truncation of their protein products; this trend has been also reported in other cohort studies [2, 3, 33]. Interestingly, the rates of missense/truncated variants in *SGCA-SGCD* may be related to the roles of each protein subunit in the formation and function of the sarcoglycan complex. Recent advances in the prediction of protein structures using Alphafold have allowed us to consider the pathogenicity of identified variants in the context of whole protein structures [34, 35] (Fig. 2). α-Sarcoglycan has a globular domain in its central extracellular region and missense variants in *SGCA* are concentrated in this globular domain. Additionally, the central extracellular region of α-sarcoglycan has been reported to exhibit ecto-ATPase activity [36]. By contrast, the extracellular regions of β-, γ-, and δ-sarcoglycans are predicted to have simple structures, without globular domains. The majority of variants in *SGCB*, *SGCG*, and *SGCD* cause protein truncation. Our previous model of sarcoglycan complex formation suggested that β-, γ-, and δ-sarcoglycans make up the core of the complex, while α-sarcoglycan binds later and is primarily on the outside of the complex [37]. On the other hand, other reports demonstrated core structure of sarcoglycan complex consists of β- and δ-sarcoglycans and later incorporates α- and γ-sarcoglycan [10, 38]. Thus, amino-acid substitutions caused by missense variants in *SGCA* most probably affect the ecto-ATPase activity of α-sarcoglycan and also cause instability of the sarcoglycan complex, while truncations of β-, γ-, and δ-sarcoglycans might affect the de novo formation of the sarcoglycan complex.

Additionally, we identified two novel variants which may prevent the resulting mutated proteins from undergoing normal intracellular processing. One of these novel variants is c.38-2A > C in *SGCA*, detected in F2, which causes aberrant splicing resulting in in-frame deletion of four hydrophobic amino acids in the signal peptide of α-sarcoglycan (yellow arrowhead, Fig. 2); this deletion is predicted by iPSORT (https://ipsort.hgc.jp/) to render the signal peptide non-functional [39] [30]. The other novel variant is c.94A > G in *SGCD* in F51, which is predicted to lead to a p.K32E substitution (white arrowhead, Fig. 2). This substitution decreases the positive charges on the cytosolic side of the transmembrane domain by replacing a basic amino acid, which was predicted to influence the membrane topology of δ-sarcoglycan using WoLF PSORT (https://wolfpsort.hgc.jp/) [40]. Further structural and functional studies on mutated proteins are required to clarify the exact roles and pathogenicity of the identified variants.

## Conclusions

In conclusion, both muscle biopsy and genetic analysis are essential for accurate diagnosis of SGPs; LGMDR3 is the most common type in Japan, while LGMDR6 is very rare. Patients with SGPs share relatively few variants. Missense variants are the most common type of change in *SGCA*, while truncation mutations account for a major proportion of *SGCB*, *SGCG*, and *SGCD* variants. Tools for prediction of splicing changes are useful for the identification of pathogenic intronic variants.

## Supplementary Information

Additional file 1.Table S1. Primers use in RT-PCR.Table S2.Guide RNA-targeted sequences for long-read sequencing. Table S3. Primers for haplotype analysis.

## Data Availability

The datasets used and/or analyzed during the current study are available from the corresponding author on reasonable request.

## Abbreviations

AG: Gain of acceptor site
AL: Loss of acceptor site
DG: Gain of donor site
DL: Loss of donor site
LGMD: Limb-girdle muscular dystrophies
LTR: Long terminal repeat
MLPA: Multiplex ligation-dependent probe amplification
nt: Nucleotides
SGP: Sarcoglycanopathy
SINE: Short interspersed nuclear element
