# Supplementary material for: Profiling of pathogenic variants in Japanese patients with sarcoglycanopathy
Source: Orphanet J Rare Dis. 2025 Jan 4;20:1. doi: 10.1186/s13023-024-03521-2 (PMC11699685; doi:10.1186/s13023-024-03521-2)
Supplement: Supplementary file 1 — Additional file 1.Table S1. Primers use in RT-PCR.Table S2.Guide RNA-targeted sequences for long-read sequencing. Table S3. Primers for haplotype analysis. [file 13023_2024_3521_MOESM1_ESM.pdf]

Additional file 1

Supplementary table S1. List of primers used in RT-PCR

Muscle for analysis	Forward primer	Reverse primer
F2	ATGGCTGAGACACTCTTCTGGACTC	CTCAGGAAGGCTCAGAAACGTCTC
F3	TGAGACACTCTTCTGGACTCCTCTC	CCTTCTTTTCGGCCCTCAATGG
F25 and F26	ATACCAAGCCGAGTTCCTGGTG	CATGACATAGGCCAGCAGCAAG
F37	CAAGGGACAACAAAGCTCAGTGTAG	TTTCCACAGGGGTTGTCTGAGATTT
F53	TAACAGGTCCAAAAGCCGTAGAAG	CTCATCCCTCACCTCTCCATCTTTG

Supplementary table S2. List of targeted sequences of the guideRNA- for long-read sequencing of *SGCG* large deletions

Genetic variation	
Exon6 deletion	AAAGTGAGCCATTCGTAAGATGG<br>GTGCCCACACGGACAAGATAGGG
Whole exon deletion	GATTGGTAACTGACATACCAGGG<br>TAGGATGTCACCTTTGGTGTAGG
Exon 1-6 deletion	GATTGGTAACTGACATACCAGGG<br>GTGCCCACACGGACAAGATAGGG

PAM sequences are underlined.

Supplementary table S3. List of primers for haplotype analysis

dbSNP ID	Forward primer	Reverse primer
rs140814016	TCCACCCCCATGAGTGTATGAAAAT	CTAGGGCAATGCTGAGGAGAAATCT
rs11656409	CAGCTCAACAGGAAAGCATCTGATC	AATTCTCACCACAGACTCCAGGATG
rs74788205	TAGAAGACTTCCTGAGGGAGGGAAA	GATAAACCTGGACTTCAACCAAGCC
rs11652273	TACACTAACCCACTCCAGGACTTTG	GCAGTAAACCACCATGTCTCATGTC
rs75456035	CCACTCTCCCCTGTTTTGTTTTTGT	CAGTCTGCAAAACAACTGGACTCAT
rs62060107	TGAATCAGAAGGAACAGGAAGCTGA	CACACACACAGCCTGCACATAATAA
rs62060110	TGTTGAAGATGCTGAAAGTCCCTTC	AACTGTTCCAGCCTAATAGCCTCTC
rs4794147	ACACCCAGCCAGAAAAGTACACATA	AGGGTAACTCTGTGAGGTGATGAAA
rs4794159	CACTCTTCTTCCCAACTTCCACGAC	GTCTTTATTATGCGGGCGTTCAGTT
rs873839	GTTGAGGCTAGGACAGGTAGAGTG	CCACGGACTCATATTAACTTCTGCC
rs117252107	TAGGTGTGGAGGGAAATGGAAAGTT	CAACCACTAACACATCAGACCCTCT
rs149121305	TTGAATCAGAACCCCAAAATGGCTC	AACCAGCAAATCTATTGAAACCGGG
rs138209506	GGTAAGCTAGTGGTTTGCATGTGTT	CCTTCTCTAGCCTGCTACCATGAAT
rs557737329	TGCTGATTAGAATTGATGCTGCTGG	TCCTTACATAACCTTGCCAGACCAA
rs117293584	GCCATGAGCATTGAACAGATGAACT	TGCAGAACCGTGAGTCAATTAAACC
rs79775854	CACAGCATCACAGGACTTTGAAGAG	TCAACTCTACTCCAGTCTGAGGTCT
rs3794365	GAGGAGTGTTCTAGGGACAGACAAG	TCTTCTCATTGCTCTGTCCTGGTTT
rs7997631	ACCAATTGCAAAATGACATGGCATG	TAGAGACGGGGTTTCACTGTGTTAG
rs141190856	CCCACAAAACACCTGCTGAATAGAT	CCACGTCAACCCTACAATACCAAGT
rs7999839	AGAATACTGAGGTAGGGCCAAAAGG	AGAAGCATGGAGGTTAAGGTCTTGT
rs111597444	CTTTCCTTTAACATACCCGCCTTCG	GCGAGAGAGAGGTTTTGTTGCTAAA
rs75131193	ATATTTTCCCACGTCACTAAAGCGC	TGGGTAACGAAGATACAGCAGAAGT
rs7991949	TCCAGAGTTTACATCACGGAACACT	CGGGTAGAAGGAGGTGAGTTGTTAA
